# A phase 0 clinical trial to evaluate the neuropharmacological profile of posaconazole for glioblastoma

**DOI:** 10.1093/noajnl/vdag174

**Published:** 2026-07-02

**Authors:** Alireza Mansouri, Leonardo de Macedo Filho, Emily Tufano, Harrison Laukhuff, Brad Zacharia, Jeffrey Neighbors, Michele Green, Nicole Derosia, Dongxiao Sun, Debarati Bhanja, Kyle Tuohy, James Connor, Dawit G Aregawi, Michael Glantz, Gelareh Zadeh

**Affiliations:** Department of Neurosurgery, Penn State College of Medicine, Hershey; Department of Neurosurgery, Penn State Cancer Institute, Hershey; Department of Neurosurgery, Penn State College of Medicine, Hershey; Department of Neurosurgery, Penn State Cancer Institute, Hershey; Department of Neurosurgery, Penn State College of Medicine, Hershey; Department of Neurosurgery, Penn State College of Medicine, Hershey; Department of Neurosurgery, Penn State College of Medicine, Hershey; Department of Neurosurgery, Penn State College of Medicine, Hershey; Department of Neurosurgery, Penn State Cancer Institute, Hershey; Department of Molecular and Precision Medicine, Penn State College of Medicine, Hershey; Department of Neurosurgery, Penn State College of Medicine, Hershey; Department of Neurosurgery, Penn State Cancer Institute, Hershey; Department of Molecular and Precision Medicine, Penn State College of Medicine, Hershey; Department of Neurosurgery, Penn State College of Medicine, Hershey; Department of Neurosurgery, Penn State Cancer Institute, Hershey; Department of Molecular and Precision Medicine, Penn State College of Medicine, Hershey; Department of Neurosurgery, Penn State College of Medicine, Hershey; Department of Neurosurgery, Penn State Cancer Institute, Hershey; Department of Neuroscience and Experimental Therapeutics, Penn State College of Medicine, Hershey; Department of Neurosurgery, NYU Langone, New York; Department of Neurosurgery, Penn State College of Medicine, Hershey; Department of Neurosurgery, Penn State College of Medicine, Hershey; Department of Neurosurgery, Penn State College of Medicine, Hershey; Department of Neurosurgery, Penn State College of Medicine, Hershey; Department of Neurosurgery, Mayo Clinic, Rochester

**Keywords:** blood-brain barrier, clinical trials, drug repurposing, GBM, Phase 0

## Abstract

**Background:**

Glioblastoma (GBM) is the most common malignant primary brain tumor and is associated with a poor prognosis. Repurposed posaconazole (PCZ) has demonstrated efficacy *in vitro* by targeting key metabolic pathways. The aim of this study was to determine the neuropharmacological profile of PCZ in patients with GBM through a Phase 0 first-in-human trial for this indication (NCT04825275).

**Methods:**

We conducted an open-label, non-randomized, parallel-arm trial in participants with primary or recurrent GBM. Patients in the treatment arm received oral PCZ for 7-10 days presurgically to achieve steady-state concentrations. Microdialysis catheters were implanted to measure interstitial drug levels. Postoperative tissue and fluid samples were analyzed to evaluate PCZ pharmacokinetics (mass spectrometry) and pharmacodynamics, including vascular density, apoptosis, and metabolite concentrations (lactate and pyruvate) compared to controls.

**Results:**

The trial was closed early due to slow accrual, enrolling 2 participants in the PCZ arm and three in the control arm. No drug-related safety issues were observed. Although PCZ was undetectable in microdialysate fluid, it accumulated in tumor tissue, with concentrations showing an association with CD31-measured endothelial content. PCZ-treated tumors exhibited trends toward lower BCL2 expression indicative of increased apoptosis, and significantly lower pyruvate levels in the tumor periphery compared to controls. Plasma lactate levels in treated patients normalized to control levels over 24 h.

**Conclusions:**

Despite limited accrual, this trial provides the first clinical evidence that oral PCZ accumulates in human GBM tissue and shows preliminary signals of metabolic modulation. These findings provide biological rationale warranting further clinical investigation, potentially utilizing direct tissue sampling rather than microdialysis.

Key Points

- PCZ accumulated in GBM tissue, significantly reducing peripheral pyruvate.
- Trends of higher apoptosis and lower lactate validate metabolic modulation.
- Results support further clinical investigation without microdialysis.

Importance of the Study

Glioblastoma relies on aerobic glycolysis, a metabolic vulnerability targeted by posaconazole in preclinical models. However, confirming blood-brain barrier penetration and target engagement in humans is essential before large-scale efficacy trials. This Phase 0 study provides the first clinical evidence that oral posaconazole accumulates in GBM tissue and shows preliminary signals of metabolic modulation, evidenced by significantly reduced peripheral pyruvate. By demonstrating the translational potential of metabolic targeting with azoles, this work establishes a biological rationale for further clinical investigation. Additionally, the study identifies that microdialysis is unsuitable for monitoring this highly protein-bound agent, refining future trial designs. These findings support further clinical investigation, including potential studies focusing on survival endpoints, and offer a verified, repurposed therapeutic candidate for this lethal malignancy.

Glioblastoma (GBM, WHO Grade 4 glioma) is the most common and deadly brain cancer in adults.^1^ As part of numerous evolutionary mechanisms to enhance their survival, GBM tumor cells undergo reprogramming of their energy metabolism to survive in hypoxic conditions.^2^ This is preferentially mediated through glycolysis over oxidative phosphorylation, leading to the production of high levels of lactate and pyruvate while consuming very little oxygen.^3^ This is in part mediated through elevated levels of hexokinases (HK1, HK2, and HK3), which catalyze the first committed step in glucose metabolism.^4^^,^^5^ The HK2 isoform also inhibits apoptosis.^6^^,^^7^ Also known as the Warburg effect, this may be a metabolic vulnerability that can be exploited therapeutically.^2^^,^^8^

*In silico* drug screening strategies have proposed the role of repurposed azoles as a therapeutic measure targeting this pathway. In addition to its impact on tumor metabolism, *in vivo* studies have shown extended survival of xenograft mice bearing intracranial GBM, a reduction of mitotic activity, and reduction of lactate levels in the tumor following treatment with PCZ.^9^ PCZ is FDA-approved for management of central nervous system fungal infections, along with an established safety and drug dosing profile, making it an ideal drug repurposing candidate. Window-of-opportunity (WOO) and Phase 0 trial designs have emerged as established tools in neuro-oncology for evaluating drug penetration and target engagement in human brain tumor tissue prior to large-scale efficacy studies, providing critical translational data before advancing to Phase 2 trials.^10^^,^^11^

However, prior to embarking on a large-scale prospective clinical trial to evaluate the efficacy of PCZ in GBM, it is critical to establish brain tumor penetrance at currently established doses while also confirming on-target pharmacodynamic properties. Establishing the neuro-pharmacokinetic profile of PCZ would also add valuable information for future dose-escalation strategies, if necessary.

Accordingly, we designed a first-in-human Phase 0 clinical trial for this indication to evaluate accumulation of PCZ in brain tumor tissue, establish its neuro-pharmacokinetic profile, and to evaluate its pharmacodynamic impact. Microdialysis catheters (MDCs) were utilized for evaluation of PCZ and metabolites in the brain interstitial fluid over time. This trial was registered under the NCT identifier NCT04825275.

## Methods

### Study Design and Ethics

We conducted an open-label, non-randomized, parallel-arm Phase 0 clinical trial at the Penn State Cancer Institute. The protocol was approved by the Institutional Review Board (IRB) and conducted in accordance with the Declaration of Helsinki. All participants provided written informed consent. Please refer to our published protocol for comprehensive details.^12^

### Participant Selection

Adults (18 years or older) with recent contrast-enhanced MRI findings suggestive of GBM with need for surgical resection. Only participants who, in the opinion of the managing surgeon, were stable enough not to require surgery sooner than 7 days were approached. Our target was to enroll 10 evaluable participants (PCZ group [*n* = 5] and control group [*n* = 5]). Detailed eligibility criteria are outlined in the previously published protocol.^12^

### Dosing and Intervention

Participants in the PCZ arm took PCZ 300 mg orally twice on study day 1, followed by 300 mg orally once daily thereafter. PCZ is known to achieve steady state in plasma at approximately 130-155 h following the first dose (5 half-lives).^13^ The surgery was scheduled such that participants in the PCZ arm received the drug for 168-240 h (7-10 days). To maintain steady-state concentrations, PCZ was administered pre-operatively on the morning of surgery at least 3-4 h prior to incision, and in the morning of postoperative day 1 (*t* = 0 for PK purposes), provided that postoperative computed tomography (CT) ruled out complications and the MDC(s) was correctly positioned.

Surgery was conducted according to standard of care for participants in both control and PCZ arms. Upon confirmation of GBM diagnosis, the research samples were prepared for assessment of intra-tumoral PCZ concentration (PCZ arm only) and other PK/PD parameters detailed in protocol and below. Tissue samples were acquired from the center of the tumor and from the tumor edge (periphery) to account for possible heterogeneity in blood-brain barrier disruption. Specific to the PCZ arm, blood samples were obtained at the time of tumor sampling, as well as to determine and compare tumor: blood PCZ concentration ratios.

Following completion of surgical resection for both cohorts, 1 MDC catheter (M Dialysis AB, Stockholm, Sweden) was placed in the tissue surrounding the tumor resection cavity, in accordance with pre-operative MRI. We used MD71 MDC catheters, maximum pore size of 20 kDa, supplied by MDilaysis. All participants had complete resection of enhancing tumor in this trial. Prior to administration of the last dose of PCZ and collection of any serial samples, a postoperative CT scan was performed to confirm the position of the MDC tips and to rule out complications (Figure S1). Dialysate sample collection began on the morning of postoperative day 1, at the same time as the last dose of PCZ administration (for the PCZ arm) to annotate “Time 0”. Up to 9 samples were obtained over 24 h from the MDC pump system. Blood samples were collected from an arterial line at the same intervals as dialysate collection.

### Study Outcomes

*Primary Outcomes:* To establish the neuro-PK profile of PCZ in people with primary or recurrent GBMs requiring open surgical resection, using microdialysis catheters (MDC).

*Secondary Outcomes:* (i). To evaluate tolerability of preoperative steady-state dosing of PCZ; (ii) to evaluate concentration of PCZ in tumor samples from the intervention cohort; and (iii) to evaluate PD properties of PCZ. The latter was analyzed based on PCZ’s effect on: Tumor Tissue—Proliferation (Ki-67 index), apoptosis (Bcl-2 assay), and vascular density (CD31 staining) in resected tissue; Fluid Biomarkers**—**Lactate and pyruvate concentrations in dialysate and plasma samples; and PK/PD Correlation—The correlation of the PCZ concentration-time profile with that of lactate and pyruvate.

### Study Setting

Participants were prospectively recruited at a single tertiary cancer center (Penn State Cancer Institute in Hershey, PA). The total duration of study participation was 14 days following the surgical resection of the tumor.

### PCZ Concentration Analyses

#### Chemicals

Posaconazole standards (purchased from Sigma-Aldrich, Missouri, USA) were prepared in methanol and stored in −20°C before use. Formic acid was purchased from J. T. Baker (New Jersey, USA). Optima LC-MS grade water, acetonitrile and methanol and other chemicals were purchased from Fisher Chemicals (New Jersey, USA).

#### Sample preparation

Posaconazole standard working solution was prepared from stock solution, by serial dilution of a posaconazole standard with methanol, resulting in concentrations from 5 to 5,000 ng/ml. The standard samples were prepared by spiking 4 ml mixed standard working solutions (5-5,000 ng/ml) into 10 ml control sample (plasma or CSF), and after vortexing, 26 ml acetonitrile: methanol: H_2_O (40: 40: 20) was added to extract the analytes from samples. Proteins were precipitated by vortexing with subsequent centrifugation at 21,130 *g* for 5 min at 4°C. The supernatant was taken and loaded to the UPLC-MS-MS system, with final concentrations of 0.5-500 ng/ml.

#### Tumor samples

Fresh frozen tumor tissue samples were gently thawed on ice. A minimum of 5 mg was placed in a 15 ml conical tube with 250 µl of Dulbecco’s Phosphate Buffered Saline (DPBS) and thoroughly pipet mixed. Samples were vortexed to ensure breakdown of tissues. Samples were briefly spun in a benchtop centrifuge and incubated for 30 min at room temperature. About 250 µl of chloroform was added with pipet mixing to each sample, followed by a 15-min incubation at room temperature. Samples were mixed and incubated for an additional 15 min at room temperature. About 200 µl of the bottom chloroform layer was transferred to 1.5 ml Eppendorf tubes, and evaporation was performed with a nitrogen evaporator. Dried samples were stored at −20°C. Standards were made by spiking 5 µl posaconazole standard working solution into 45 µl acetonitrile: methanol: H_2_O (40: 40: 20) and vortexed before loading ontothe UPLC-MS-MS system.

#### Plasma/dialysate samples

About 10 µl samples were extracted with 30 µl acetonitrile: methanol: H_2_O (40: 40: 20). The plasma or dialysate samples were vortexed before being centrifuged under the same conditions as those for the standard samples, and the supernatant was loaded onto the UPLC-MS-MS system. The calculated concentrations from the standard curves were adjusted (multiplied by 4 for a dilution factor of 4) during extraction to reflect the posaconazole *in vivo* levels in plasma or CSF.

### UPLC-MS-MS Analysis

Posaconazole was analyzed using a Sciex QTRAP 6500+ mass spectrometry coupled with a Sciex Exion UPLC separation system. A 1.7 µm Acquity UPLC BEH C18 analytical column (2.1 × 100 mm, Waters, Ireland) was used to separate posaconazole from other impurities. The gradient elution was conducted using a flow rate of 0.3 ml/min with the following conditions: initiate in 5% mobile phase B (acetonitrile) and 95% solvent A (0.1% acetic acid in water) and hold for 0.5 min, then a linear gradient to 95% mobile phase B over 2 min, and keep the 95% mobile phase B for 1.5 min to flush the column before back to initial conditions to equilibrate the column.

The Sciex QTrap 6500+ mass spectrometer was equipped with an electrospray ionization probe operated in positive mode. The decluster potential (DP) was 84 V for posaconazole; the entrance potential (EP) was 10 V, the collision energy (CE) was 47 V for posaconazole; the collision cell exit potential (CXP) was 27.6 V for posaconazole. The curtain gas (CUR) was 35 l/h, and the collision gas (CAD) was medium. The ion spray voltage was 5,500 V, the temperature was 550°C, gas 1 was 25 l/h, and gas 2 was 25 l/h.

The multiple reaction monitoring mode (MRM) was used to analyze and quantify posaconazole, with the transitions of *m/z* 701 > 683 for posaconazole. All peaks were integrated and quantified by Sciex OS 3.0 software.

### Pharmacodynamic Analyses

#### Evaluation of plasma lactate and pyruvate

Lactate and pyruvate levels in the plasma were determined using ELISA kits (Human Lactate Dehydrogenase D: LSBio LS-F51793; Human Pyruvate Dehydrogenase Phosphatase: LSBio LS-F32415) per the manufacturer’s instructions. Plasma was diluted 1:10 in the provided sample diluent. Briefly, a total of 100 µl of each sample or standard was added to a 96 well pre-coated plate and incubated for 90 min (pyruvate) or 2 hours (lactate) at 37°C. The samples were removed, and 100 µl of 1× biotinylated detection antibody was incubated at 37°C for 1 h. After washing 3× with 1× wash buffer, 100 µl of 1× HRP-Streptavidin conjugate was added to each well for 30 min at 37°C for 1 h. The plate was then washed 5× with 1× wash buffer, followed by 90 µl per well of 3′,3′,5′,5′-tetramethylbenzidine (TMB) substrate for 10-20 min at 37°C. Finally, 50 µl per well of stop solution was added to stop the reaction. Optical density was measured at 450 nm using a microplate reader (SpectraMax Gemini EM plate reader; Molecular Devices).

#### Evaluation of biological impact in tumor tissue

Tumor tissue homogenates were lysed in NP40 buffer (50 mM Tris-HCl (pH 7.4), 150 mM NaCl, 1% NP40, and 5 mM EDTA) with a protease inhibitor cocktail). Samples were homogenized manually, sonicated, and centrifuged at 12,000 rpm for 15 min at 4°C. Total protein concentrations in the supernatant were determined using the Bradford protein determination assay. Tissue samples were flash-frozen in liquid nitrogen within 10 min of resection and stored at −80°C prior to analysis to minimize metabolite degradation.

##### Apoptosis

Apoptosis was assessed using a BCL2 ELISA kit (Human BCL2: LSBio LS-F4134) per the manufacturer’s instructions. Equal protein concentrations (300 µg) were used for evaluation. Briefly, 100 µl of each sample or standard was added to a 96 well pre-coated plate and incubated for 1 h at 37°C. Samples were removed, and the plate was incubated with 100 µl of Detection Reagent A for 1 hour at 37°C. The plate was washed 3 times with 1× wash buffer, followed by a 30-min incubation with 100 µl of 1× Detection Reagent B at 37°C. A final wash step was performed 5 times before adding 90 µl of TMB substrate for 10-20 min and 50 µl of stop solution. Optical density was determined using a microplate reader at 450 nm. BCL2 protein quantification by ELISA provides continuous numerical output from whole tissue homogenate, avoiding inter-observer variability of semi-quantitative IHC, and has been validated against IHC for BCL2 measurement in tumor tissue.^14^

##### Vascularity

CD31/PECAM-1 was used to evaluate endothelial cell content as a quantitative surrogate for vascular density, using an ELISA kit (Human PECAM-1/CD31: LSBio LS-F5247) per the manufacturer’s instructions. Equal protein concentrations (200 µg) were used for evaluation. Briefly, 100 µl of each sample or standard was added to a 96-well pre-coated plate and incubated for 1 h at 37°C. Samples were aspirated, followed by a 1-h incubation with Detection Reagent A at 37°C. Plates were washed 3 times with 1× wash buffer, incubated with Detection Reagent B for 30 min at 37°C, and washed 5 times again. About 90 µl of TMB substrate was incubated for 10-20 min, followed by stop solution. Optical density determination is described above.

##### Pyruvate and lactate

Lactate and pyruvate levels in the tumors were determined using ELISA kits (listed above; Human Lactate Dehydrogenase D: LSBio LS-F51793; Human Pyruvate Dehydrogenase Phosphatase: LSBio LS-F32415). Equal concentrations of 3 mg and 10 µg were used for lactate and pyruvate, respectively, for evaluation. Protein determination was performed per the manufacturer’s instructions and described above.

##### Proliferation

This was based on the reported overall Ki67 value reported by the pathology department.

## Results

**Figure 1.**
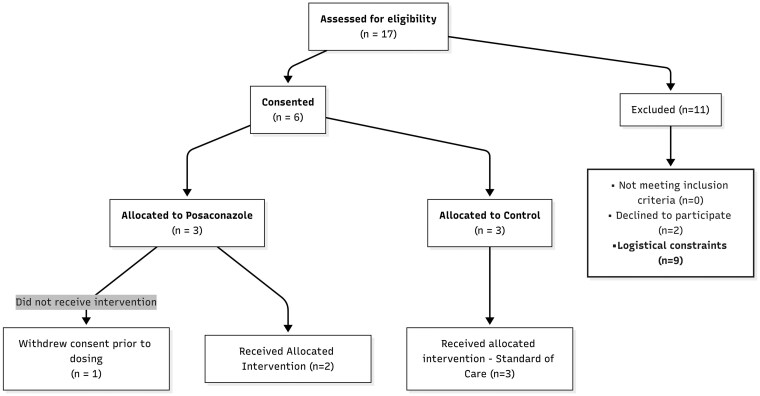
CONSORT flow diagram of participant enrollment, allocation, and analysis.

The study opened on January 27, 2022, and closed for slow accrual on February 28, 2025. Three participants were consented for the PCZ arm, and three were consented for the control arm. Demographic and tumor-specific information are outlined in Table 1. One participant in the PCZ arm withdrew consent prior to any drug administration. There were no safety events attributed to the drug. In 1 participant, the MDC could not be removed at the bedside, necessitating intraoperative removal. The CONSORT diagram can be found in Figure 1.

**Figure 2.**
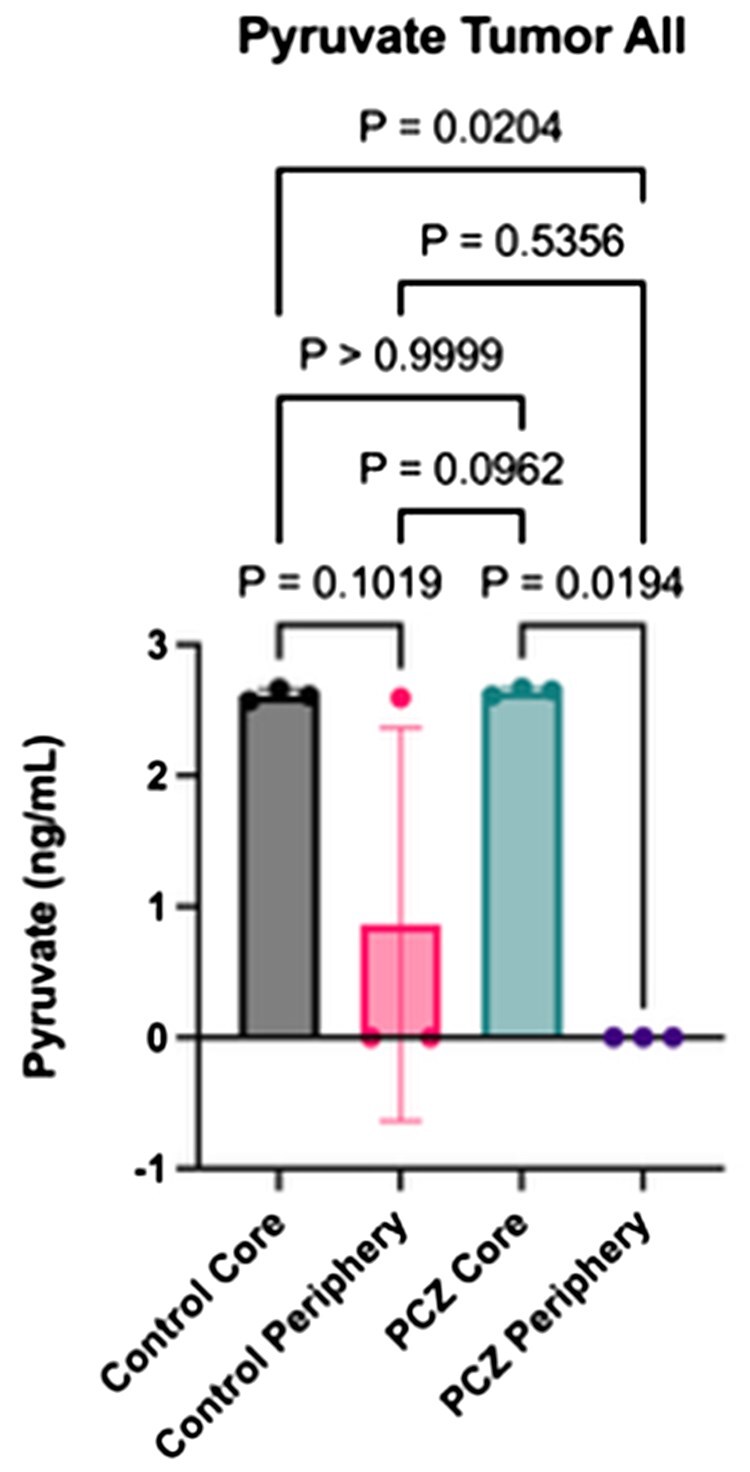
Pyruvate concentration in the tumor microenvironment before and after intervention.

**Table 1.** Individual patient demographics and clinical characteristics

Characteristic	Control 1	Control 2	Control 3	PCZ 1	PCZ 2
Age (years)	70.4	67.1	51.5	83.1	73.9
Sex	Male	Male	Male	Male	Male
Race	White	White	White	Other/mixed	White
BMI (kg/m²)	28.2	24.2	35.0	28.0	27.0
Tumor volume (cc)	32.8	6.6	54.0	49.5	12.4
GBM status	Newly diagnosed	Newly diagnosed	Newly diagnosed	Newly diagnosed	Recurrent
MGMT promoter	Unmethylated	Methylated	Unmethylated	Unmethylated	Unmethylated
TERT promoter	Pathogenic variant	Pathogenic variant	Pathogenic variant	Pathogenic variant	Pathogenic variant
EGFR status	Amplified/variant	Amplified/variant	Amplified/variant	Not detected	Amplified/variant
CDKN2A/B	Not detected	Not detected	Not detected	Deleted	Deleted
PTEN status	Not detected	Pathogenic variant	Not detected	Not detected	Pathogenic variant

### Pharmacokinetics of PCZ

The plasma concentration of PCZ over time recapitulated known pharmacokinetics associated with PCZ from other studies. Steady-state plasma concentrations were assessed from the time of surgery to 24 h post-surgery; exposure was consistent between the 2 patients with an area under the concentration-time curve (AUC_0-24 h_) of 16,745.6 and 15,673.2 ng/ml*h (Figure S2). Steady-state plasma concentrations at the time of surgery were 628 and 1,190 ng/ml for these 2 patients, respectively.

The concentration of PCZ in the dialysate fluid was below the limit of detection. In tumor tissue, however, PCZ was detectable. As expected, this was variable depending on whether samples were obtained from the core or periphery. In PCZ-002 (newly diagnosed GBM), the drug concentration was higher in the enhancing periphery compared with the necrotic core (126.7 ng/g vs. 62.7 ng/g). In PCZ-006 (recurrent GBM), however, the drug concentration was higher in the homogeneously enhancing core compared with the periphery (1254.0 ng/g vs 17.8 ng/g). In the latter participant, the substantially higher drug accumulation in the core was despite the steady-state plasma drug concentration being nearly half of that of the newly diagnosed patient (628 ng/ml vs 1190 ng/ml).

### Pharmacodynamics of PCZ

The impact of PCZ on biological endpoints was analyzed separately for tumor core and tumor periphery samples. These endpoints were compared with equivalent tumor samples from control participants (Table 2).

**Table 2.** Baseline characteristics and response biomarkers for trial participants

	PCZ1	PCZ2	Ctrl1	Ctrl2	Ctrl3
Status	New	Recurrent	New	New	New
Ki67 (%)	20	30	25	40	60
CD31 (ng/ml)	3.07	1.19	2.64	2.11	1.97
Bcl2 (pg/ml)	467.5	148	722	717	420
Lactate core (mIU/ml)	0	93.37	378.95	20.32	64.36
Pyruvate core (ng/ml)	1.31	1.34	2.60	1.33	1.39
Tumor volume (cm^3^)	49.5	12.4	32.8	6.6	54
Drug concentration core (ng/g)	62.2	1,254			
Drug concentration periphery (ng/g)	126.7	17.8			
Plasma drug concentration (ng/ml)	1190	628			
Tumor: plasma ratio (Core)	0.05	2.0			
Tumor: plasma ratio (periphery)	0.11	0.03			
Key image	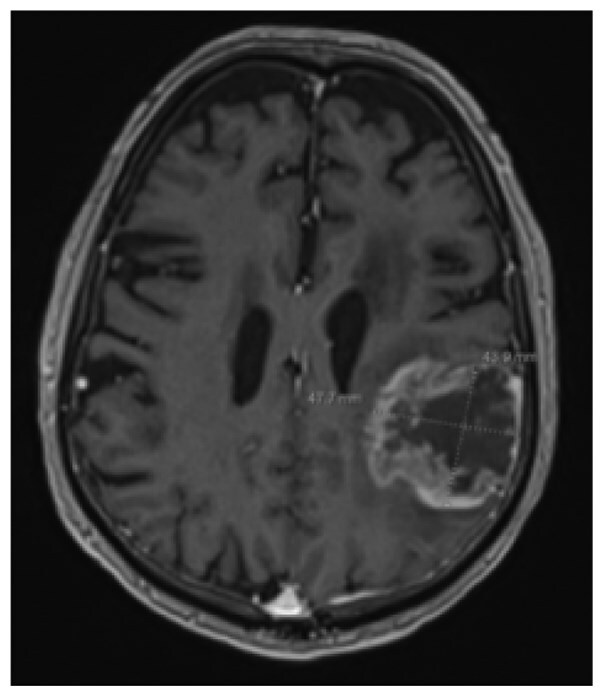	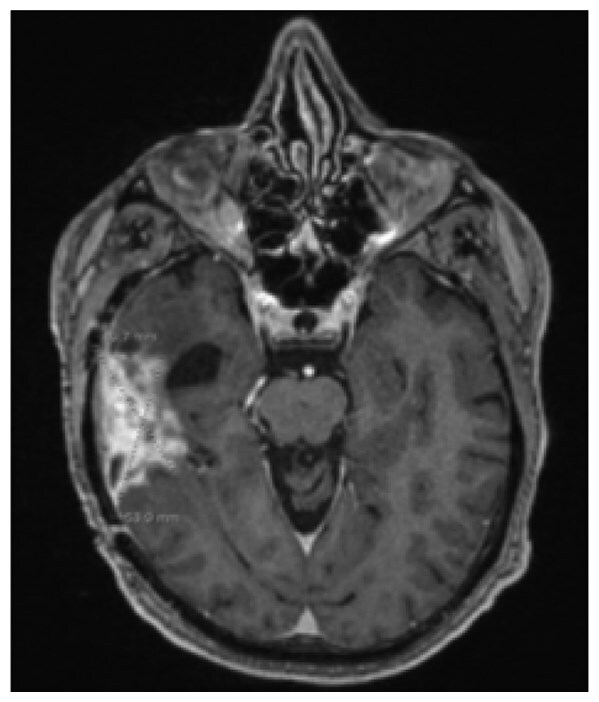	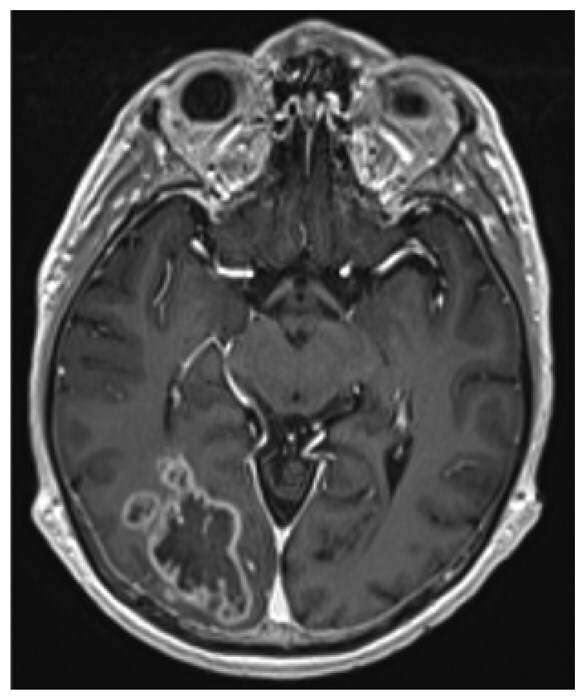	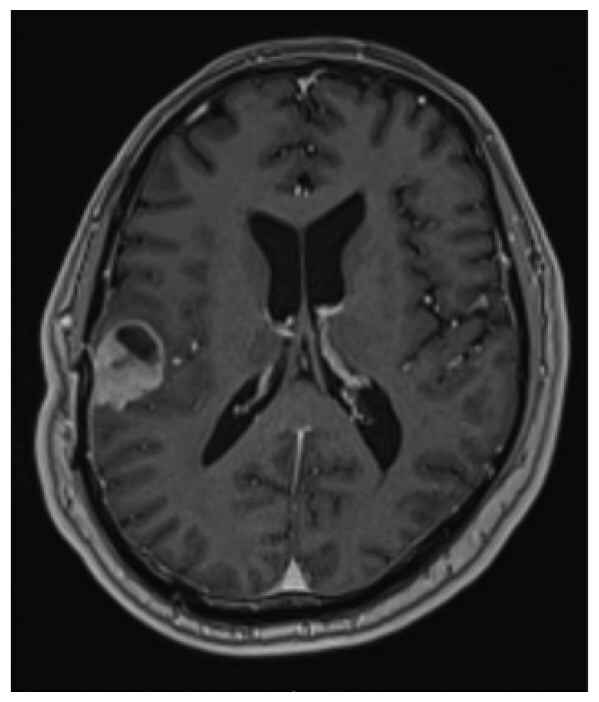	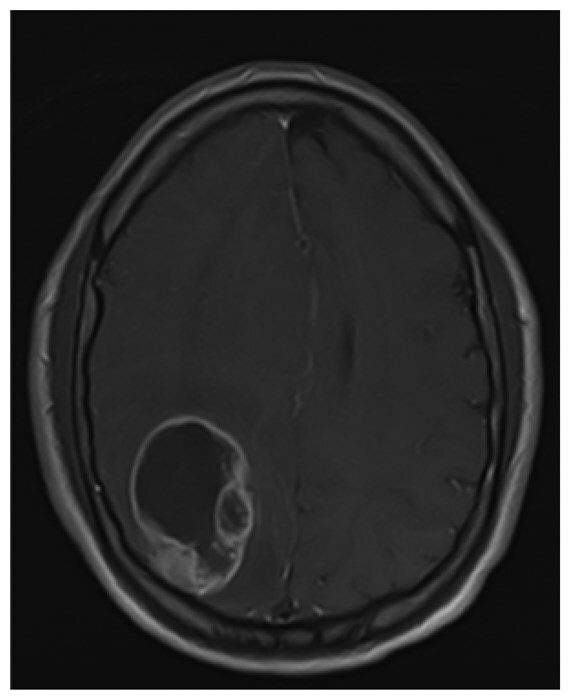

#### Apoptosis

To assess the role of PCZ treatment on apoptosis within the tumor, we measured the anti-apoptosis protein, BCL2. BCL2 expression was slightly lower in the PCZ-treated group compared to controls when combining the core and peripheral tumor samples, though this failed to reach statistical significance (536.3 vs 307.8 pg/ml, *P* = .35). When analyzing PCZ-treated tumor core and periphery levels, there was a minimal reduction of BCL2 expression when compared to the respective control tissues (core: 706.3 vs 294.5 pg/ml, *P* = .80; periphery: 366.3 vs 321.0 pg/ml, *P* = .80). These data suggest a non-significant trend towards increased apoptosis in the PCZ-treatment group compared to controls.

#### Vascularity

As an additional biological measure of the tumor vascular microenvironment, we evaluated CD31/PECAM-1, a marker of endothelial cell content. Our analysis revealed no differences in endothelial cell content when comparing PCZ-treated and control tumors (core and periphery combined) (2.24 vs 2.13 ng/ml, *P* = .91), or in the core (2.36 vs 2.12 ng/ml, *P* = .80) or periphery (2.12 vs 2.14 ng/ml, *P* > .99) when analyzed separately.

#### Pyruvate and lactate

Next, we examined the impact of PCZ on metabolic measures of tumor growth. Lactate and pyruvate are both used as a source of energy to fuel the hallmark rapid proliferation seen in GBM. Pyruvate was below the limit of detection for all peripheral samples from PCZ-treated patients and 66% of the peripheral samples from control patients, and this difference reached significance in the PCZ group (2.65 vs 0 ng/ml, *P* = .02, Figure 2). In contrast, pyruvate was detectable in the core of control and PCZ-treated patients, and no significant differences in levels were observed when comparing the 2 (2.62 vs 2.65 ng/ml, *P* > .99). There was a slight trend towards decreased lactate levels in PCZ-treated samples compared to control in the combined group (154.5 vs 46.7 mlU/ml), but this failed to reach significance in the core (115.0 vs 62.6 mlU/ml, *P* > .99) and the periphery (194 vs 111.17 mlU/ml, *P* = .80). These findings suggest that PCZ treatment may modestly impact tumor metabolism, particularly in the periphery, though overall metabolic measures were limited.

Elevated plasma pyruvate and lactate can reflect a large range of conditions, including certain cancers. While we measured pyruvate and lactate in the tumors directly, we also evaluated the impact of PCZ treatment on circulating levels over time. Interestingly, we observed that plasma LDH levels were higher at time 0 in control patients compared to PCZ-treated patients (2,593.4 mIU/ml vs 259.5 mIU/ml, respectively) but normalized by 24 h among the 2 arms (1229.5 mIU/ml vs 1198.3 mIU/ml, respectively). In contrast, plasma pyruvate levels did not follow this pattern, showing that control patients exhibited higher pyruvate only between 30 min and 2 h post-surgery. At all other time points, pyruvate levels were slightly higher in PCZ-treated patients compared to controls.

## Discussion

Our promising *in vivo* data on the efficacy of repurposed PCZ for GBM was the impetus behind this Phase 0 clinical trial. Based on the notion that the human blood-brain and blood-tumor barrier structure and physiology differ from that of mice,^9^ we saw the need to confirm sufficient drug penetration into brain and tumor tissue, with concordant proof of biological concept, prior to launching into advanced-phase efficacy trials. Despite closing early due to poor accrual, we observed accumulation of PCZ in tumor tissue with a potential association with tumor endothelial content and preliminary signals of impact on tumor metabolism.

### Drug Concentration in Plasma, Tumor, and Dialysate Fluid

The plasma concentration and half-life measurements for PCZ in our study recapitulated known pharmacokinetic parameters associated with PCZ, and no drug-related adverse events were noted. While PCZ was detectable in tumor tissue, the concentration varied significantly across the 2 participants and between core and periphery. Specifically focusing on the regions with the highest drug concentration, PCZ concentration was 10-fold higher in the homogeneously enhancing core of a recurrent GBM compared with that of the enhancing periphery of a newly diagnosed GBM (1,254.0 ng/g vs 126.7 ng/g). In the prior *in vivo* study upon which this trial was based, at a dosing regimen of 25 mg/kg, the median concentration of PCZ ranged from 500 ng/g to just over 1000 ng/g of tumor tissue (in GSC8-18 and U87 xenograft models,^9^ respectively). Drug concentrations showed an exploratory association with CD31-measured endothelial content, suggesting possible accumulation via regions of disrupted vasculature. While no definitive conclusions can be drawn based on two participants, it is conceivable that inherent differences between the vasculature of tumor stem cells vs. mature cells, and their integrity following chemoradiation, may be a possible explanation for these differences. This should be taken into consideration when designing eligibility criteria (primary vs recurrent GBM) for future follow-up trials.

The dosing regimen used in our trial (300 mg BID on day 1, followed by 300 mg daily) was based on the drug brochure recommended dosing for prophylaxis against CNS infections. Based on established dose conversion factors between animals and humans, the mouse dosing used in our *in vivo* study would translate to approximately 2 mg/kg or ∼140 mg for a 70 kg adult.^15^ Therefore, the current dose was likely necessary but perhaps not sufficient. A recent Phase 1 dose escalation study evaluated the addition of mebendazole, part of the azole family of antifungals, to temozolomide in newly diagnosed GBM.^16^  The authors noted reversible Grade 3 derangement of liver enzymes at the highest dose level (200 mg/kg/day), which was reversible upon lowering the dose or drug discontinuation. Therefore, a dose escalation study may be warranted to evaluate the maximum tolerated dose.

The failure to detect PCZ in the microdialysate warrants detailed mechanistic consideration. PCZ is approximately 98% albumin-bound, meaning that only the small unbound fraction (∼2%) is available for passive diffusion across the semipermeable membrane of the MD71 microdialysis catheter (molecular weight cutoff: 20 kDa). At the steady-state plasma concentrations observed in our patients (628-1,190 ng/ml), the predicted unbound fraction would be approximately 13-24 ng/ml—a concentration at or below the lower limit of detection of our mass spectrometry assay. This finding is consistent with published experience: even temozolomide, which has substantially lower protein binding (∼15%), achieves brain: plasma AUC ratios of only approximately 18% in GBM patients as measured by intracerebral microdialysis.^17^ Burns and colleagues have further highlighted that the standard semipermeable membrane microdialysis is fundamentally limited for highly lipophilic, protein-bound agents.^18^ Critically, the absence of PCZ in the dialysate does not reflect a failure of CNS drug delivery—direct mass spectrometry of tumor homogenate confirmed substantial tissue accumulation in both treated patients, with the recurrent GBM patient achieving concentrations within and exceeding the preclinical therapeutic range. This dissociation between ECF detection failure and confirmed tissue accumulation underscores that plasma pharmacokinetics and direct intraoperative tissue sampling provide more pharmacologically meaningful data for highly protein-bound lipophilic agents than ECF microdialysis. On the basis of these findings, we recommend that future trials of PCZ abandon MDC as a pharmacokinetic endpoint in favor of intraoperative tissue sampling from multiple tumor regions. While MDC placement was well-tolerated in this series, with 1 catheter requiring intraoperative rather than bedside removal, the procedural burden contributed to patient hesitancy during enrollment, and the unfavorable risk-benefit ratio of MDC for this drug class does not justify its use in future PCZ studies.

### Pharmacodynamic Effects

While potential trends supporting the pharmacodynamic effect of PCZ were observed, these were by no means significant. This can be attributed to the short interval of drug administration (7-10 days prior to surgery). The aforementioned Phase 1 trial and a more recent Phase 2 trial of mebendazole + temozolomide vs mebendazole + lomustine in recurrent GBM,^19^ administered the antifungal throughout the adjuvant phase of treatment. The pharmacodynamic endpoints employed in this study were selected based on the established mechanism of PCZ through HK2 inhibition. Future studies would benefit from incorporating hypothesis-free approaches such as bulk tumor metabolomics or single-nucleus transcriptomics on paired pre- and post-treatment samples, as demonstrated in recent perioperative and window-of-opportunity trial designs.^20^^,^^21^

#### Reasons for poor accrual and lessons learned

The study failed to accrue the target number of participants. Factors likely affecting this poor accrual included most participants requiring urgent surgery (sooner than the requisite 7-10 days for preoperative dosing), concerns regarding the safety of MDCs, and the lack of therapeutic intent for the trial. The goal of implementing the use of MDCs in this Phase 0 trial was to conduct a real-time assessment of the brain microenvironment, enabling measurement of PCZ concentration and its PK. This was based on the rationale that plasma analysis is not informative for evaluating drug delivery across the BBB and that intracerebral monitoring through MDCs would allow for sampling the brain interstitial fluid for approximate measurements. However, this would only be necessary if no drug concentration was noted in tumor tissue. While steady-state dosing ensures the maximal possible concentration of drug in tumor tissue, numerous window-of-opportunity trials have demonstrated that a shorter interval prior to surgery may suffice for drug detection and evaluation of on-target pharmacodynamics.^22^^,^^23^ Contemporary Phase 0/2 trigger designs in recurrent GBM have demonstrated that preoperative dosing windows as short as 4-7 days are feasible, that direct intraoperative tissue sampling provides pharmacologically informative data, and that PK-threshold-based graduation criteria can efficiently identify patients most likely to benefit from continued treatment while limiting unnecessary drug exposure.^10^^,^^24^^,^^25^ The perioperative matched-biopsy design reported by Drummond et al further confirms that patients not requiring urgent surgery are willing to undergo complex perioperative protocols when the scientific rationale is clearly communicated.^20^ Accordingly, a Phase 1/2 design focusing on recurrent GBM is most appropriate. The minimum number of days for preoperative dosing may be shortened while targeting steady-state concentrations. The Phase 1 component would involve dose escalation to identify the maximum tolerated dose. Participants demonstrating sufficient tumor drug accumulation by intraoperative tissue sampling would continue onto the Phase 2 component and receive daily PCZ. Confirmation of drug accumulation in the tumor prior to Phase 2 continuation represents an incremental improvement over recently published azole trials and would limit unnecessary participant exposure. Multi-site enrollment should also be considered to address the accrual challenges encountered at a single center.

## Conclusion

Despite not meeting accrual goals, the findings of this trial demonstrate accumulation of PCZ in tumor tissue with preliminary signals of biological activity, and no drug-related safety events. Together with limited drug-drug interactions associated with PCZ and standard-of-care chemotherapy regimens in GBM, these findings provide biological rationale for further clinical investigation of PCZ as a concomitant adjuvant drug, warranting a thoughtfully designed subsequent trial.

## Supplementary Material

vdag174_Supplementary_Data

## Data Availability

The data underlying this article will be shared on reasonable request to the corresponding author.
