# Supplementary figures and images for: A phase 0 clinical trial to evaluate the neuropharmacological profile of posaconazole for glioblastoma
Source: Neurooncol Adv. 2026 Jul 2;8(1):vdag174. doi: 10.1093/noajnl/vdag174 (PMC13394498; doi:10.1093/noajnl/vdag174)

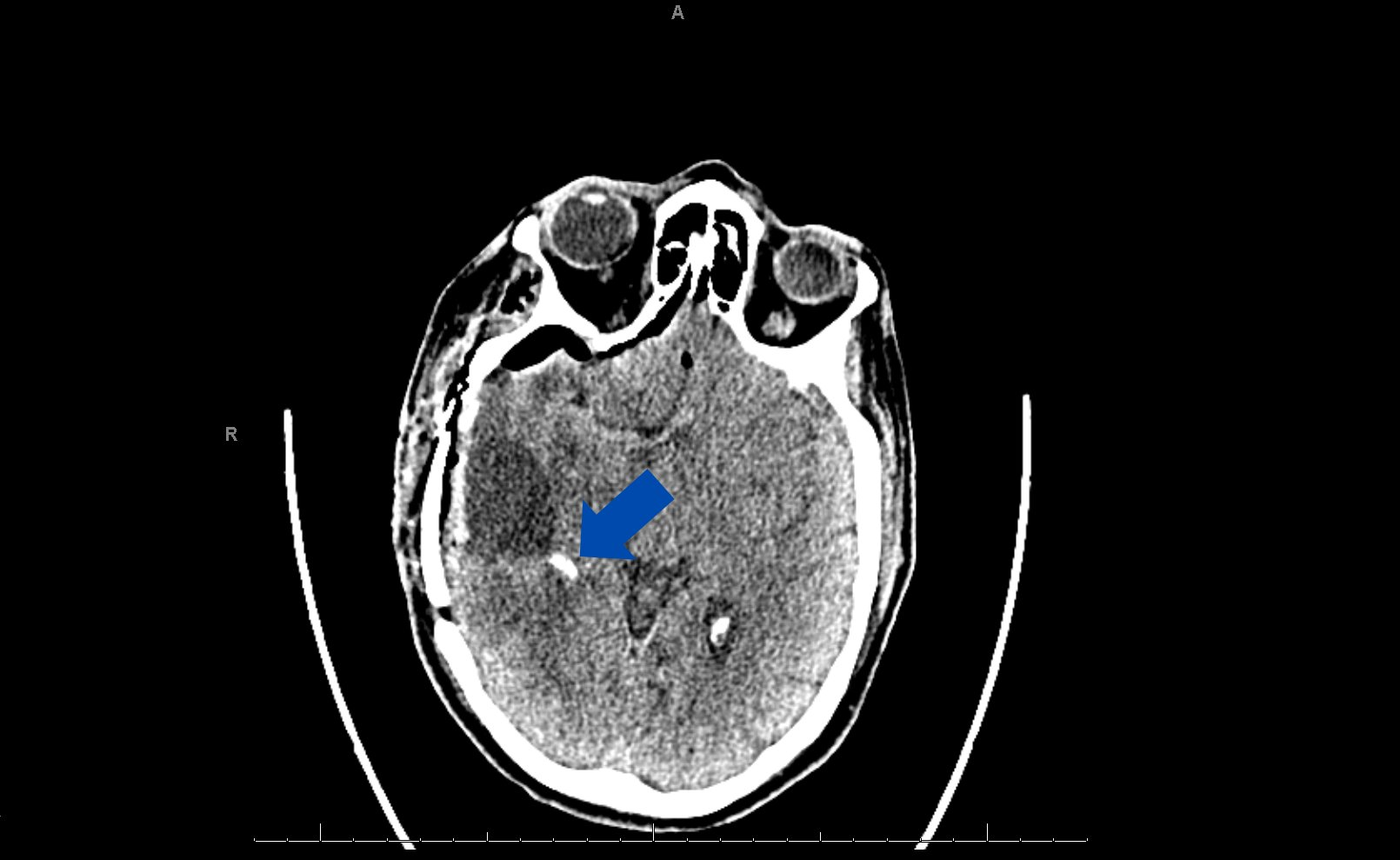

Supplement: vdag174_Supplementary_Data [file vdag174_supplementary_data.zip › Figure_S1.tif]

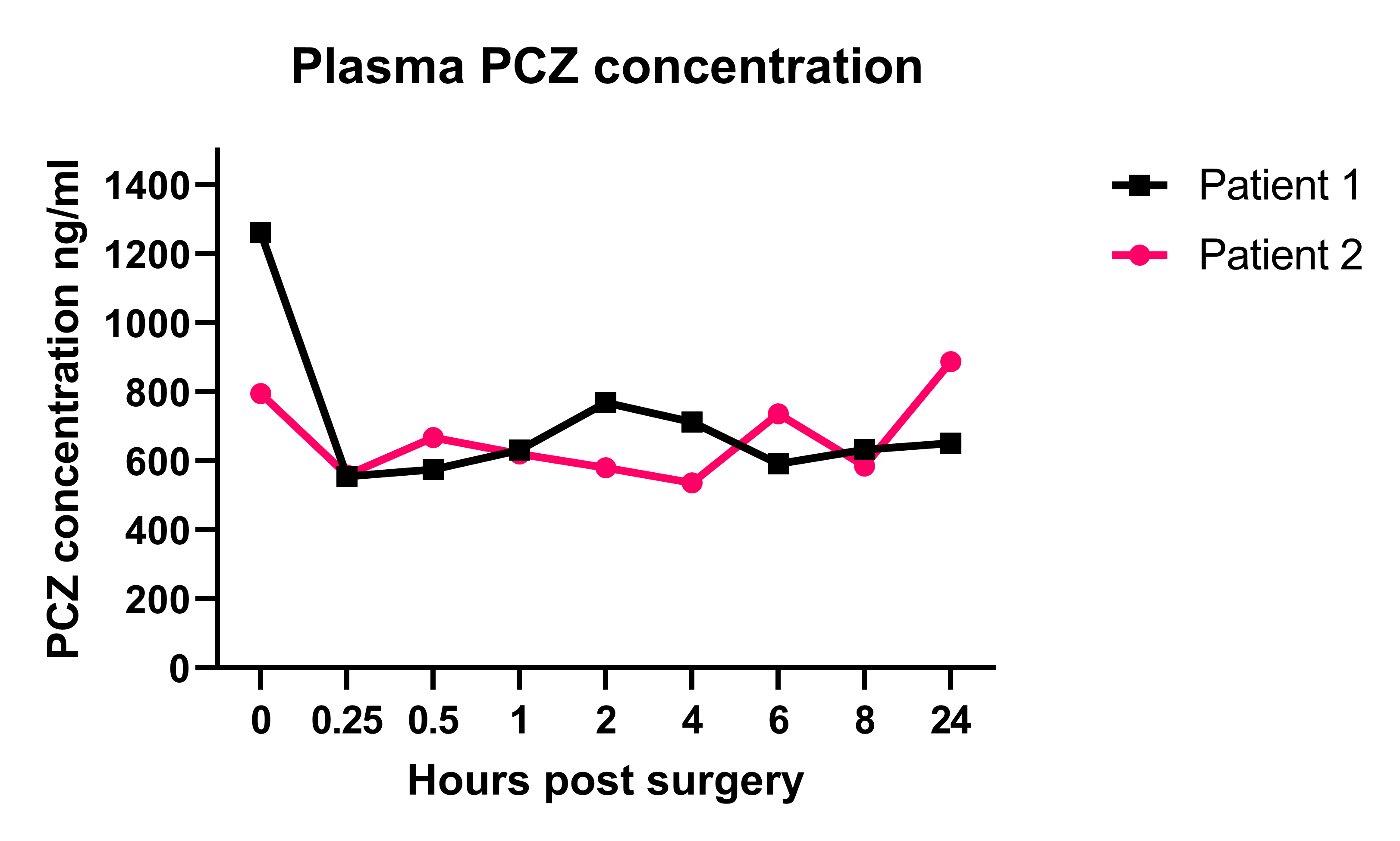

Supplement: vdag174_Supplementary_Data [file vdag174_supplementary_data.zip › Figure S2.tif]
